# Mixed antagonistic effects of the ginkgolides at recombinant human ρ_1_ GABA_C_ receptors

**DOI:** 10.1016/j.neuropharm.2012.06.067

**Published:** 2012-11

**Authors:** Shelley H. Huang, Trevor M. Lewis, Sarah C.R. Lummis, Andrew J. Thompson, Mary Chebib, Graham A.R. Johnston, Rujee K. Duke

**Affiliations:** a Discipline of Pharmacology, School of Medical Sciences, Faculty of Medicine, University of Sydney, Australia; b School of Medical Sciences, University of New South Wales, Australia; c Department of Biochemistry, University of Cambridge, Cambridge, United Kingdom; d Faculty of Pharmacy, University of Sydney, Australia

**Keywords:** Ginkgolide, Bilobalide, Mixed-antagonism, Use-dependent, GABAρ receptor, GABA_C_ receptor, ρ_1_ GABA_C_ receptor, *Xenopus* oocytes, nACh, nicotinic acetylecholine, 5-HT, 5-hydroxytryptamine/serotonin, GABA, γ-aminobutyric acid, GA, ginkgolide A, GB, ginkgolide B, GC, ginkgolide C, PTX, picrotoxinin, RDL, resistance to dieldrin

## Abstract

The diterpene lactones of *Ginkgo biloba*, ginkgolides A, B and C are antagonists at a range of Cys-loop receptors. This study examined the effects of the ginkgolides at recombinant human ρ_1_ GABA_C_ receptors expressed in *Xenopus* oocytes using two-electrode voltage clamp. The ginkgolides were moderately potent antagonists with IC_50_s in the μM range. At 10 μM, 30 μM and 100 μM, the ginkgolides caused rightward shifts of GABA dose–response curves and reduced maximal GABA responses, characteristic of noncompetitive antagonists, while the potencies showed a clear dependence on GABA concentration, indicating apparent competitive antagonism. This suggests that the ginkgolides exert a mixed-type antagonism at the ρ_1_ GABA_C_ receptors. The ginkgolides did not exhibit any obvious use-dependent inhibition. Fitting of the data to a number of kinetic schemes suggests an allosteric inhibition as a possible mechanism of action of the ginkgolides which accounts for their inhibition of the responses without channel block or use-dependent inhibition. Kinetic modelling predicts that the ginkgolides exhibit saturation of antagonism at high concentrations of GABA, but this was only partially observed for ginkgolide B. It also suggests that there may be different binding sites in the closed and open states of the receptor, with a higher affinity for the receptor in the closed state.

## Highlights

► Ginkgolide A, B and C were found to be mixed antagonists at ρ_1_ GABA_C_ receptors. ► The ginkgolides were not use-dependent. ► The ginkgolides required channel activation by GABA for dissociation. ► Kinetic modelling predicted an allosteric mechanism of action for the ginkgolides. ► The model predicted binding with higher affinity to a closed-state of the receptor.

## 1 Introduction

**Fig. 1:**
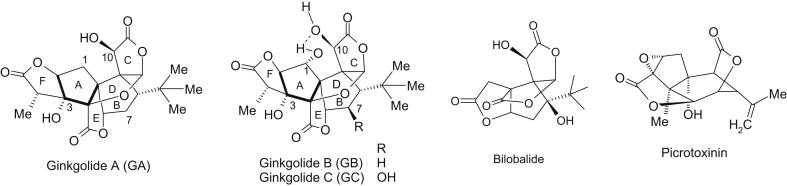
Structures of ginkgolides A, B and C (GA, GB and GC) (20 carbon atoms), bilobalide (15 carbon atoms) and picrotoxinin (PTX) (15 carbon atoms). These compounds have cavity-like structures made up of a highly oxygenated carbon skeleton, including two lactone rings and an epoxy group in PTX, and three lactone rings in bilobalide and ginkgolides. The lipophilic side chain (isopropenyl group in PTX and *t*-butyl group in bilobalide and ginkgolides) is attached to the underside of the cavity. Ginkgolides A, B and C have two common hydroxyl groups at C3 and C10 as shown in ginkgolide A. The hydroxyl group at C10 is located at the top and the C3 hydroxyl underneath the main cage comprising rings F, A, D and C. Two additional rings E and B project outward from the main cage. The C3 hydroxyl of the ginkgolides is next to the polar lactone ring E whereas the only hydroxyl group in PTX is located at the nonpolar underside of the cage. Note that ginkgolide A lacks the hydroxyl at C1 which acts as an H-bond donor to the C10 hydroxyl in ginkgolides B and C. The C7 in ginkgolide C projects upwards to the oxolane ring D.

Ginkgolides A, B and C (GA, GB and GC; Fig. 1) are diterpene lactones and, along with the sesquiterpene lactone bilobalide (Fig. 1), are found in the leaves of the *Ginkgo biloba* tree. The extract of *G. biloba* leaves has been used for treatments of cerebral and peripheral vascular dysfunctions and neurosensory disorders (Blumenthal et al., 2000). Generally, the Ginkgo leaf extract is standardized to contain 5–7% terpene lactones, consisting of 2.8–3.4% ginkgolides A, B and C, and 2.6–3.2% bilobalide (Blumenthal et al., 2000). With their oxygenated cage-like structure and a lipophilic side chain, bilobalide and ginkgolides bear structural resemblance to the chloride channel blocker picrotoxinin (PTX, Fig. 1) and they also block GABA_A_ and insect GABA_RDL_ receptors and glycine receptors in a similar manner to PTX (Ivic et al., 2003; Huang et al., 2003, 2004; Hawthorne et al., 2006; Heads et al., 2008; Jensen et al., 2010; Thompson et al., 2012). At lower potency, PTX also blocks the nicotinic acetylcholine (nACh) and 5-hydroxytryptamine (type 3, 5-HT_3_) 5-HT_3_ receptors (Erkkila et al., 2004; Das and Dillon, 2005; Thompson et al., 2011). There is evidence that the binding sites of ginkgolides, bilobalide and PTX are similarly located to that of PTX at glycine, GABA_RDL_, and 5-HT_3_ receptors (Hawthorne et al., 2006; Heads et al., 2008; Thompson et al., 2011, 2012).

GABA_A_, GABAρ (GABA_C_) and GABA_RDL_ and glycine receptors are anion-selective, and nACh and 5-HT_3_ receptors are cation-selective members of the Cys-loop receptor superfamily. Receptors in this superfamily mediate fast synaptic transmission and are appropriately located in the CNS to perform specific functions. GABA_A_ receptors are present in all CNS regions (Olsen and Sieghart, 2009) and GABA_C_ receptors largely in the retina (Qian and Ripps, 2009). Glycine receptors are mainly found in the brain stem and spinal cord (Yevenes and Zielhofer, 2011). GABA_RDL_ receptors are found in the invertebrate brain (Ffrench-Constant et al., 1993).

The receptors in the Cys-loop superfamily have a pentameric arrangement of subunits forming a central ion-conducting pore. Each of the subunits has a large extracellular N-terminal domain that contributes to the agonist binding site and a very short extracellular C-terminus. The N- and C-termini are connected by four consecutive helical transmembrane domains (M1–M4) of which M2 lines the channel pore (Miyazawa et al., 2003). The opening of the channel involves movement of the M2 domains. The intracellular loop linking M1 to M2 (M1–M2 linker) is important for ion selectivity and an extracellular loop linking M2 to M3 (M2–M3 linker) is critical for signal transduction of agonist binding to the M2 domain for channel opening (Barry and Lynch, 2005; Miller and Smart, 2010).

The GABA_C_ receptor is composed of ρ subunits of which three (ρ_1_–ρ_3_) have been cloned from mammalian retinal cDNA libraries. The different ρ subunits are able to form homooligomeric receptors or heterooligomeric receptors when expressed in *Xenopus* oocytes.

Co-expression of the ρ subunit with the GABA_A_ γ subunit forms a receptor with functional properties closely similar to a GABA_C_ receptor in retinal bipolar cells (Feigenspan and Bormann, 1994, 2006; Qian and Ripps, 2009). The major GABA_A_ receptors are heterooligomeric 2:2:1 assemblies of different isoforms and splice variants of the α, β, γ subunit (Olsen and Sieghart, 2009), whereas the invertebrate GABA_RDL_ receptor is a homooligomeric assembly of the RDL subunit (Ffrench-Constant et al., 1993). The glycine receptors are homooligomeric assemblies of different isoforms of the α subunits or heterooligomeric assemblies the α and β subunits (Yevenes and Zielhofer, 2011).

The subunits of the Cys-loop receptors have high amino acid sequence homology in the M2 domains. The degree of homology is greater when considering just the anion- or cation-selective receptor subunits and greater again for each receptor subtype. The M2 residues are numbered from 0′ to 20′ denoting the intracellular to extracellular positions. The M2 residues in the subunits are generally highly conserved with the exception of the residue at position 2′. In the GABA_C_ receptors, this residue is proline in the ρ_1_ subunit, and serine in the ρ_2_ and ρ_3_ subunits. The ρ_2_ subunit has been shown to confer insensitivity of the GABA_C_ receptors to PTX (Enz and Bormann, 1995; Zhang et al., 1995; Carland et al., 2008). The residue 2′ of the GABA ρ subunits influences the response kinetics, receptor pharmacology, ion selectivity, and conductance of GABA_C_ receptors (Zhang et al., 1995; Qian et al., 1999; Wotring et al., 2003, 2008; Carland et al., 2004a,b,; Filippova et al., 2004; Qian and Ripps, 2009; Zhu et al., 2007).

We have previously shown that ginkgolides A, B and C noncompetitively block GABA-mediated chloride currents with slightly lower potency to bilobalide and PTX at recombinant human α_1_β_2_γ_2L_ GABA_A_ receptors; and bilobalide exhibits mixed-type noncompetitive antagonism and use-dependent action similar to PTX at recombinant human ρ_1_ GABA_C_ receptors (Huang et al., 2003, 2004, 2006). Here we extend the study of these cage compounds by examining the effects of ginkgolides A, B and C on recombinant human ρ_1_ GABA_C_ receptors expressed in *Xenopus* oocyte.

## 2 Material and methods

### 2.1 Materials

Human ρ_1_ GABA_C_ receptor subunit cDNA subcloned into pcDNA 1.1 (Invitrogen, San Diego, CA, USA) was kindly provided by Dr. George Uhl (National Institute for Drug Abuse, Baltimore, MD, USA). GABA and DMSO were purchased from Sigma Chemical Co. (St Louis, MO, USA). Ginkgolide A, B and C were isolated from the 50:1 *G. biloba* leaf extract purchased from Winshing (Australia) Pty Ltd. and purified by recrystallization following short column chromatography and. The ^1^H and ^13^C NMR spectra of the purified picrotoxinin and the ginkgolides were consistent with the published data (Perry et al., 2001; van Beek, 2005), and also indicated purity >98% in all cases. Drug solutions were prepared by diluting 100 mM aqueous stock solution of GABA and either 100 mM or 200 mM DMSO stock solutions of ginkgolides A, B and C in ND96 (96 mM NaCl, 2 mM KCl, 1 mM MgCl_2_·6H_2_O, 1.8 mM CaCl_2_, 5 mM HEPES, pH 7.5). The highest concentration of DMSO superfusing the oocytes was 0.8% at which concentration DMSO had no effects.

### 2.2 Expression of ρ_1_ GABA_C_ receptors in *Xenopus laevis* oocytes

Female *X. laevis* were anaesthetized with 0.17% ethyl 3-aminobenzoate in saline and a lobe of the ovaries surgically removed. The lobe of ovaries was rinsed with a low chloride Ringer's solution (OR-2 buffer) that contained 82.5 mM NaCl, 2 mM KCl, 1 mM MgCl_2_.6H_2_O, 5 mM HEPES, pH 7.5, and treated with collagenase A (2 mg/mL in OR-2, Boehringer Mannheim, Germany) for 2 h to separate oocytes from connective tissues and follicular cells. Released oocytes were then thoroughly rinsed in ND96 buffer supplemented with 2.5 mM sodium pyruvate, 0.5 mM theophylline and 50 μg/ml gentamycin, and stage V–VI oocytes were collected.

Human ρ_1_ GABA_C_ receptor subunit cDNA was linearized using the restriction enzyme *Xba* 1 and transcribed using the “mMESSAGE mMACHINE” kit (Ambion Inc., Austin, TX, USA). RNA (10 ng/50 nl) was injected into the cytoplasm of defollicated oocytes using a 15–20 μm diameter tip glass micropipette Nanoject injector (Drummond Scientific Co., Broomali, PA, USA). The oocytes were incubated for 2–7 days at 18 °C in ND96 buffer with a twice-daily change of buffer.

### 2.3 Electrophysiological recording

Whole-cell currents from homomeric ρ_1_ GABA_C_ receptors expressed in oocytes were recorded using two-electrode voltage clamp. Recording microelectrodes were fabricated with a micropipette puller (Narishige Scientific Instrument Lab, Tokyo, Japan) and filled with 3 M KCl solution. Oocytes were clamped at −60 mV and continuously superfused with ND96 buffer. The currents elicited in response to the application of GABA were recorded using a GeneClamp 500 amplifier (Axon Instruments, now Molecular Devices, Sunnyvale, CA, USA), digitized with a Mac Lab 2e recorder (AD Instruments, Sydney, NSW, Australia) and Chart (version 3.5.2) software (AD Instruments) on a Macintosh Quadra 605 computer. A test dose of 10 μM GABA was applied to each oocyte to confirm receptor expression prior to the start of each experiment. For inhibition curves, a range of ginkgolide concentrations were co-applied with 0.5 μM (∼EC_15_ GABA), 1.2 μM (∼EC_50_), 3 μM (∼EC_80_) or 10 μM (∼EC_100_) test concentrations of GABA in ND96 buffer. Co-applications of ginkgolides were interleaved with test applications of GABA alone to monitor the response throughout the experiment. For GABA concentration–response curves, a range of GABA concentrations (0.01 − 100 μM) were applied and interleaved with co-applications in the presence of a fixed concentration of ginkgolide (10 μM, 30 μM or 100 μM). In this way, a full concentration–response curve was obtained for GABA in the presence and absence of one concentration of a ginkgolide from each oocyte. A washout period of 3–5 min was allowed between each application.

### 2.4 Analysis of data

Analysis and curve fitting was performed using GraphPad Prism v3.02 (GraphPad Software, San Diego, California, USA). Concentration–response curves were assembled from the peak currents recorded from the range of applied GABA concentrations, both in the presence and absence of ginkgolides. The data were expressed as a percentage of the averaged maximum current (*I*_max_) and fitted by least squares non-linear regression with the empirical Hill equation

$${I/I}_{\max}={\left[A\right]}^{n_{\text{H}}}/\left({\text{EC}}_{50}^{n_{\text{H}}}+{\left[A\right]}^{n_{\text{H}}}\right)$$

where [*A*] is the agonist concentration, *n*_H_ is the Hill coefficient and EC_50_ is the effective concentration that evoked a 50% of *I*_max_ response. Similarly, inhibition curves were assembled from the peak currents recorded from the range of ginkgolide concentrations applied in the presence of a fixed concentration of GABA. The data were expressed as a percentage of the peak current (*I*_max_) obtained from the application of the GABA concentration alone. The concentration that inhibited 50% of *I*_max_ (IC_50_) was estimated from fitting the data with the Hill equation, where the concentration of the ginkgolide is substituted for the agonist concentration. Unless otherwise noted, parameters were calculated from individual oocytes and then averaged.

The concentration–response curves were fitted with the different mechanisms of inhibition using a least squares non-linear regression in GraphPad Prism. Each data set consisting of a control GABA concentration–response curve and three curves in the presence of ginkgolide (10, 30 and 100 μM) were all fitted simultaneously with the same set of parameters. The values for *K*_a_ and *E* were fixed at 3.2 μM and 11, respectively (see Results).

The statistical significance of differences between GABA responses with and without antagonists was determined by two-way ANOVA method, whereas the differences between IC_50_ values at different GABA concentrations and the differences in levels of inhibition between peak and steady-state GABA responses, by Students *t*-test at the significance level of *P* < 0.05.

### 2.5 Homology modelling and docking

Using FUGUE, the protein sequence for the human ρ_1_ GABA_C_ receptor subunit (accession number P24046) was aligned with the GLIC subunit, a bacterial member of the nAChR superfamily of cationic ion channels activated by protons (PDBID, 3EAM; Shi et al., 2001). GLIC was chosen as it is homomeric, it is reported as being in the open configuration and it has a higher resolution (2.9 Å) than the nAChR structure (2BG9). A three-dimensional homology model was generated using MODELLER 9v8 (Sali and Blundell, 1993) and the best model selected by Ramachandran plot using RAMPAGE (Lovell et al., 2003). The three-dimensional structures of ginkgolide A (GA), ginkgolide B (GB), ginkgolide C (GC) were extracted from the Cambridge Structural Database (reference codes FUGTOQ01, FATYOO and UFEHES, respectively) and solvent molecules removed.

Rigid ligand docking into the wild type ρ_1_ GABA_C_ receptor homology model was carried out using GOLD 3.0 (The Cambridge Crystallographic Data Centre, Cambridge, UK). The binding site was defined by the C_α_ atom of T6′ on chain A and constrained to be within 10 Å of the T6′ residue of chains A and C (a region that encompassed the positions between 2′ and 9′). These amino acids were chosen based on the binding of similar compounds at other Cys-loop receptors (Enz and Bormann, 1995; Xu et al., 1995; Zhang et al., 1995; Perret et al., 1999; Zhorov and Bregestovski, 2000; Das and Dillon, 2005; Hawthorne et al., 2006; Chen et al., 2006; Erkkila et al., 2008; Heads et al., 2008; Jensen et al., 2010; Thompson et al., 2011, 2012). Ten genetic algorithm runs were performed for each ligand giving a total of 30 solutions. Ten genetic algorithm runs using default parameters were performed for each ligand giving a total of 30 solutions. Docked clusters were identified using the rms analysis implemented in GOLD. Potential hydrogen bond interactions were visualised with PyMol 1.2.

## 3 Results

### 3.1 Functional properties of ρ_1_ GABA_C_ receptors in *X. laevis* oocytes

Cytoplasmic injection of human wild-type ρ_1_ GABA_C_ receptor subunit cRNA into oocytes resulted in functional homomeric wild type human ρ_1_ GABA_C_ receptors similar to previous reports (Kusama et al., 1993; Duke et al., 2000; Goutman and Calvo, 2004; Carland et al., 2004a,b; 2008; Huang et al., 2006). Inward whole-cell currents ranged from 0.2 to 3.0 μA (at a holding potential of −60 mV) over the concentration range of 0.1–100 μM GABA. The range of GABA EC_50_ and Hill coefficient (*n*_H_) values were between 0.90–1.09 μM and 1.4–2.3 respectively (Table 1), consistent with previous reports for ρ_1_ GABA_C_ receptors. There was little if any desensitization to GABA observed upon continued application of GABA.

### 3.2 Inhibition of GABA-mediated currents

**Fig. 2:**
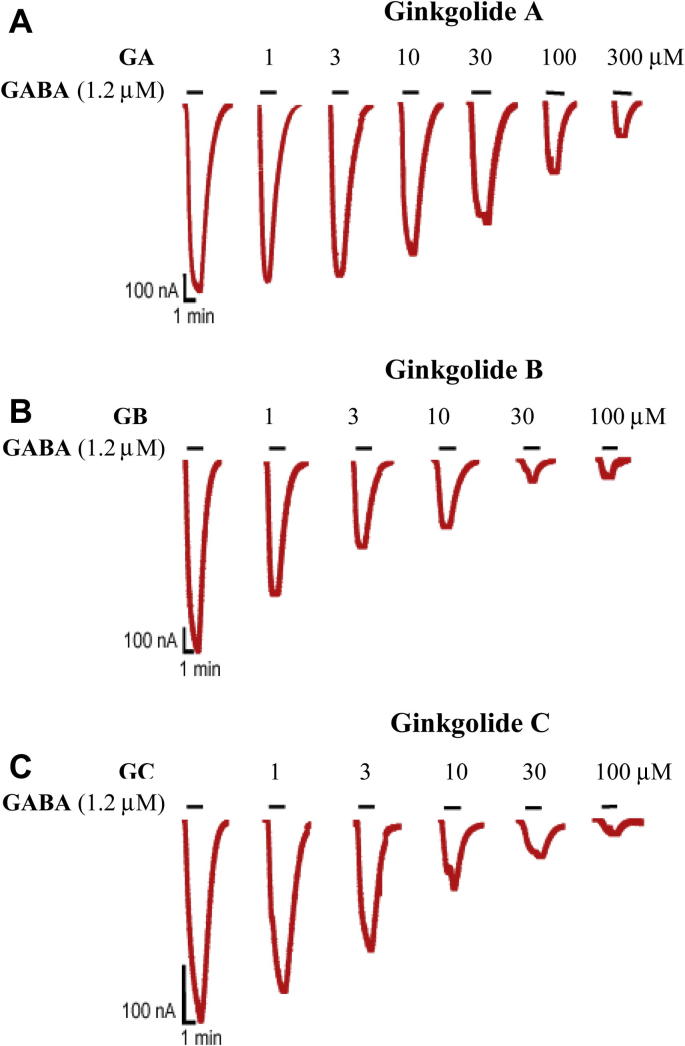
Current traces produced by 1.2 μM GABA (EC_50_, solid bar) in the presence of (**A**) ginkgolide A, (**B**) ginkgolide B and (**C**) ginkgolide C at various concentrations from human ρ1 GABA_C_ receptors expressed in *Xenopus* oocytes. The bars indicate duration of drug application. The ginkgolides did not have any effect on their own when tested at 100 μM.

**Fig. 3:**
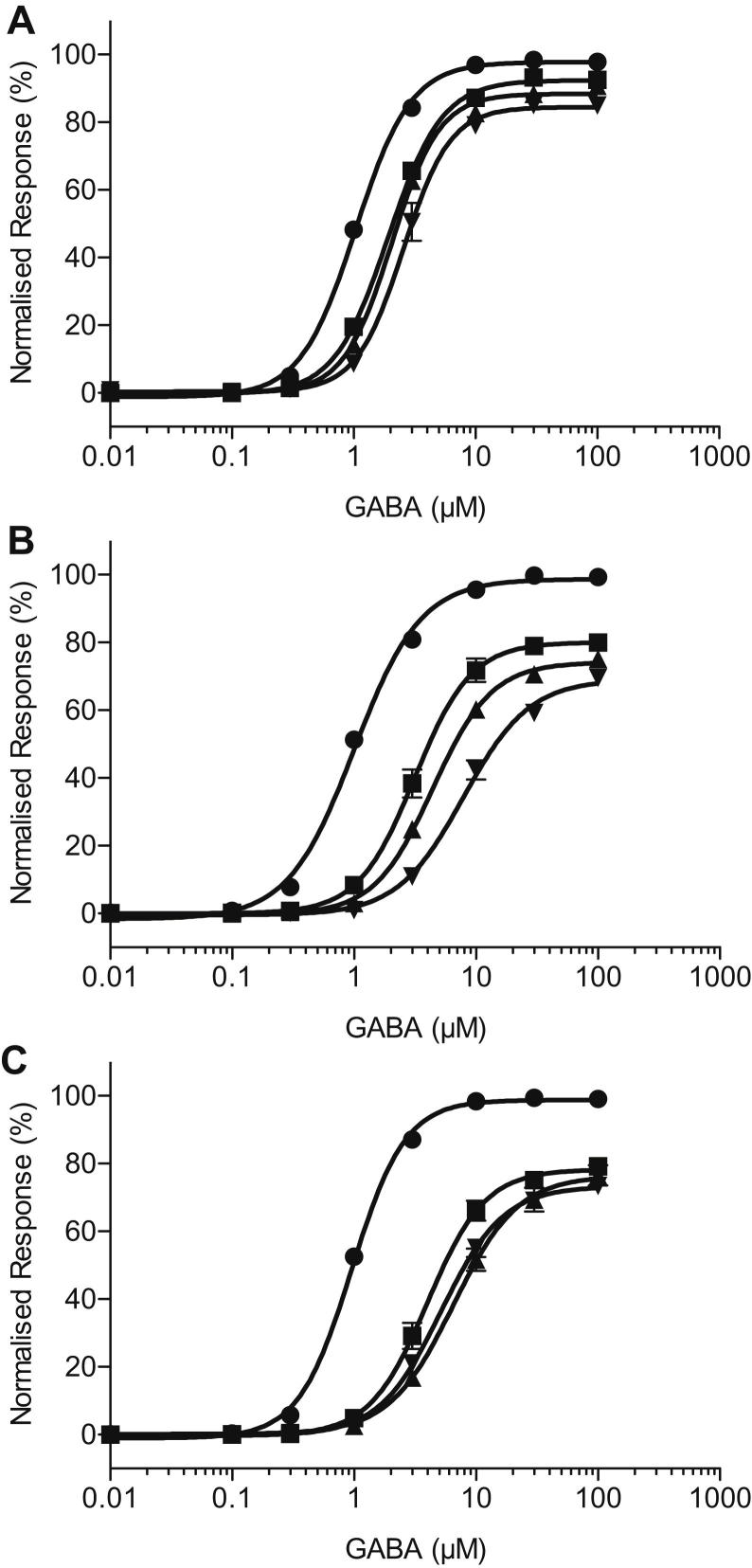
Agonist concentration–response curves for GABA, in the presence and absence of (**A**) ginkgolide A, (**B**) ginkgolide B and (**C**) ginkgolide C from recombinant human ρ_1_ GABA_C_ receptors. For each panel, the control concentration–response curve (●) is shown along with the subsequent curves obtained in the presence of 10 μM (■), 30 μM (▴) and 100 μM (▾) ginkgolide. Note the approximately parallel rightward shifts in the concentration–response curves, along with an inhibition of the maximum response with an increase in ginkgolide concentration, which is characteristic of a mixed-antagonism. The inhibition of the maximum response by ginkgolides exhibits some degree of ‘saturation’ that is most obvious for ginkgolide C. That is, there is no further inhibition of the maximum response (or a small change) with a further increase in ginkgolide concentration from 30 to 100 μM. The data are fitted with the Hill equation and the average EC_50_, Hill coefficients and normalized maximum responses estimated from these data are shown in Table 1. Data are mean ± S.E.M. (*n* = 4–6). Where the error bar is not obvious, it is entirely within the plotted symbol.

Concentration–response curves were assembled from the peak current observed upon application of GABA or co-application of GABA with ginkgolides A, B and C at 10 μM, 30 μM and 100 μM. Examples of the whole-cell currents recorded from co-application of ginkgolides are shown in Fig. 2. In each case, the ginkgolides produced approximately parallel rightward shifts in the GABA concentration–response curves and a decrease in the maximum response (Fig. 3), characteristic of mixed-antagonism. The mean values of the EC_50_, Hill coefficient (*n*_H_) and the maximum response obtained from fitting the Hill equation are shown in Table 1. A comparison of the EC_50_ values and maximum responses showed that all GABA concentration–response curves in the presence of the ginkgolides were significantly different from the corresponding GABA control curves obtained from the same oocyte (Table 1). Ginkgolide A was the least potent of the three compounds, with a 1.9-fold, 2.3-fold and 2.5-fold increase in the EC_50_ value in the presence of 10, 30 and 100 μM respectively. Ginkgolide B was the most potent, with 3.2-fold, 4.7-fold and 7.5-fold increases in the EC_50_ (for 10, 30 and 100 μM respectively). Ginkgolide C was more similar in potency to ginkgolide B, with 3.8-fold, 6-fold and 5.3-fold increases in the EC_50_ (for 10, 30 and 100 μM respectively). Ginkgolide C also exhibited no apparent further increase in the EC_50_ or a further decrease in the maximum response with an increase in the concentration from 30 to 100 μM, which is suggestive of saturation of inhibition. The ginkgolides had no effects when applied at 100 μM in the absence of GABA.

**Fig. 4:**
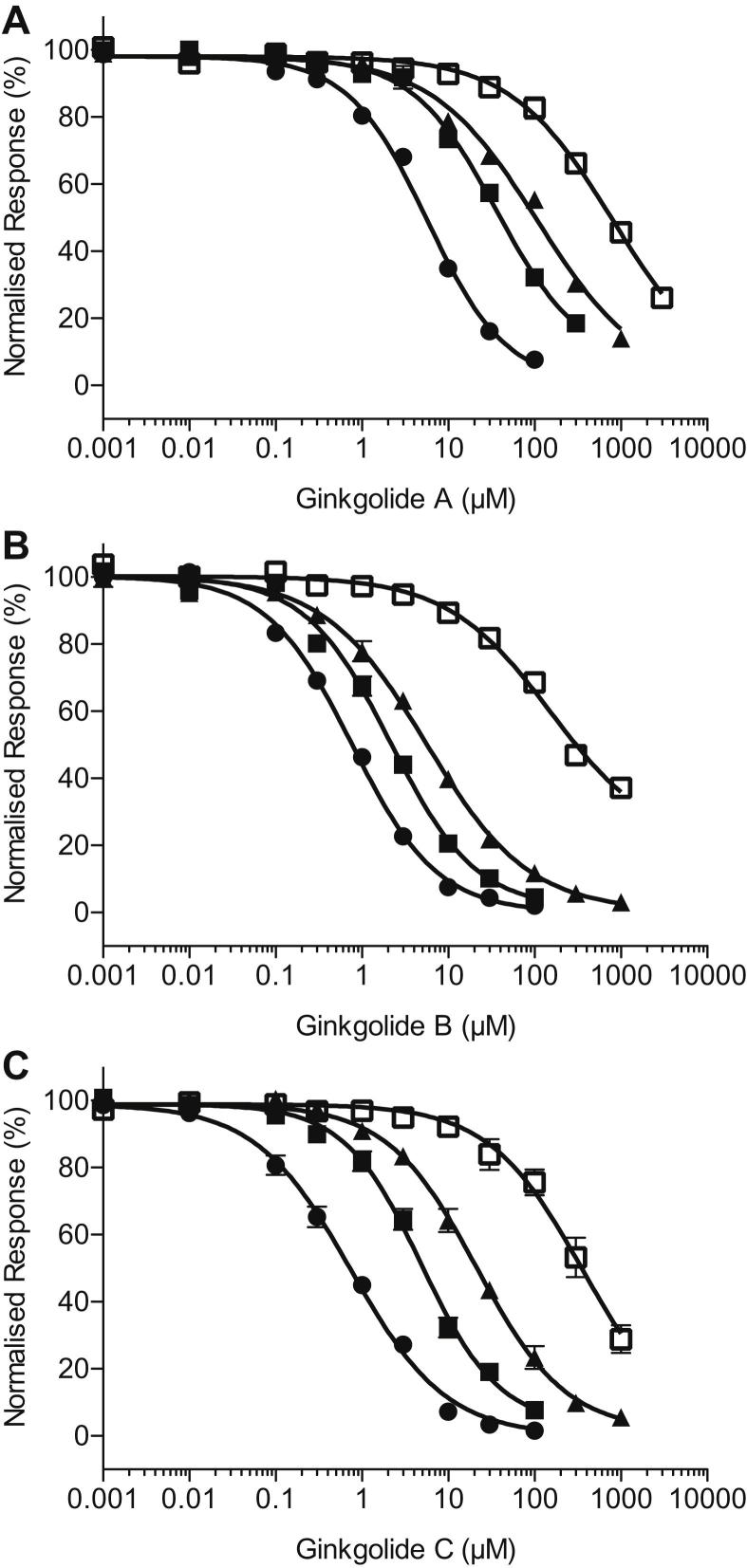
Inhibition concentration–response curves recorded from recombinant human ρ_1_ GABA_C_ receptors for (**A**) ginkgolide A, (**B**) ginkgolide B and (**C**) ginkgolide C, in the presence of 0.5 μM (●), 1.2 μM (■), 3 μM (▴) and 10 μM () GABA. The data are fitted with the Hill equation and the average IC_50_ and Hill coefficients estimated from these data are shown in Table 2. Data are mean ± S.E.M. (*n* = 5–7 oocytes). Where the error bar is not obvious, it is entirely within the plotted symbol. Note that antagonism appears to saturate at the highest concentration of ginkgolide B.

The inhibition curves assembled from the peak current responses to co-application of a fixed GABA concentration and increasing concentrations of ginkgolides A, B and C are shown in Fig. 4. The parameters obtained from fitting the data with the Hill equation are shown in Table 2. Each of the ginkgolides A, B and C exhibited increased IC_50_ values when GABA concentration was increased. For ginkgolide B, the inhibition curve in the presence of 10 μM GABA began to reach a bottom plateau of approximately 30% of *I*_max_ in five out of the seven oocytes investigated, but it was not possible to apply concentrations greater than 3 mM to try and define this bottom plateau more fully. This incomplete inhibition is a characteristic of a mechanism that exhibits saturation of inhibition (Smart and Constanti, 1986; Huang et al., 2006). Ginkgolide B was the most potent of the three ginkgolides at all GABA concentrations examined. It was approximately 7.7-, 18-, 32- and 6.7-fold more potent than ginkgolide A, and 1.1-, 2.3-, 3.8- and 4-fold more potent than ginkgolide C at 0.5 μM, 1.2 μM, 3 μM and 10 μM GABA, respectively. Using the IC_50_ values of ginkgolide B at each GABA concentration as the control for comparison, the IC_50_ values for ginkgolide A were significantly higher in each case (*P* < 0.01). The difference in the IC_50_ values between ginkgolides B and C at 0.5 μM GABA was not significant (*P* = 0.5452), but was significant at 1.2 μM, 3 μM and 10 μM GABA (*P* < 0.01).

### 3.3 Use-dependence effect of ginkgolides A, B and C

**Fig. 5:**
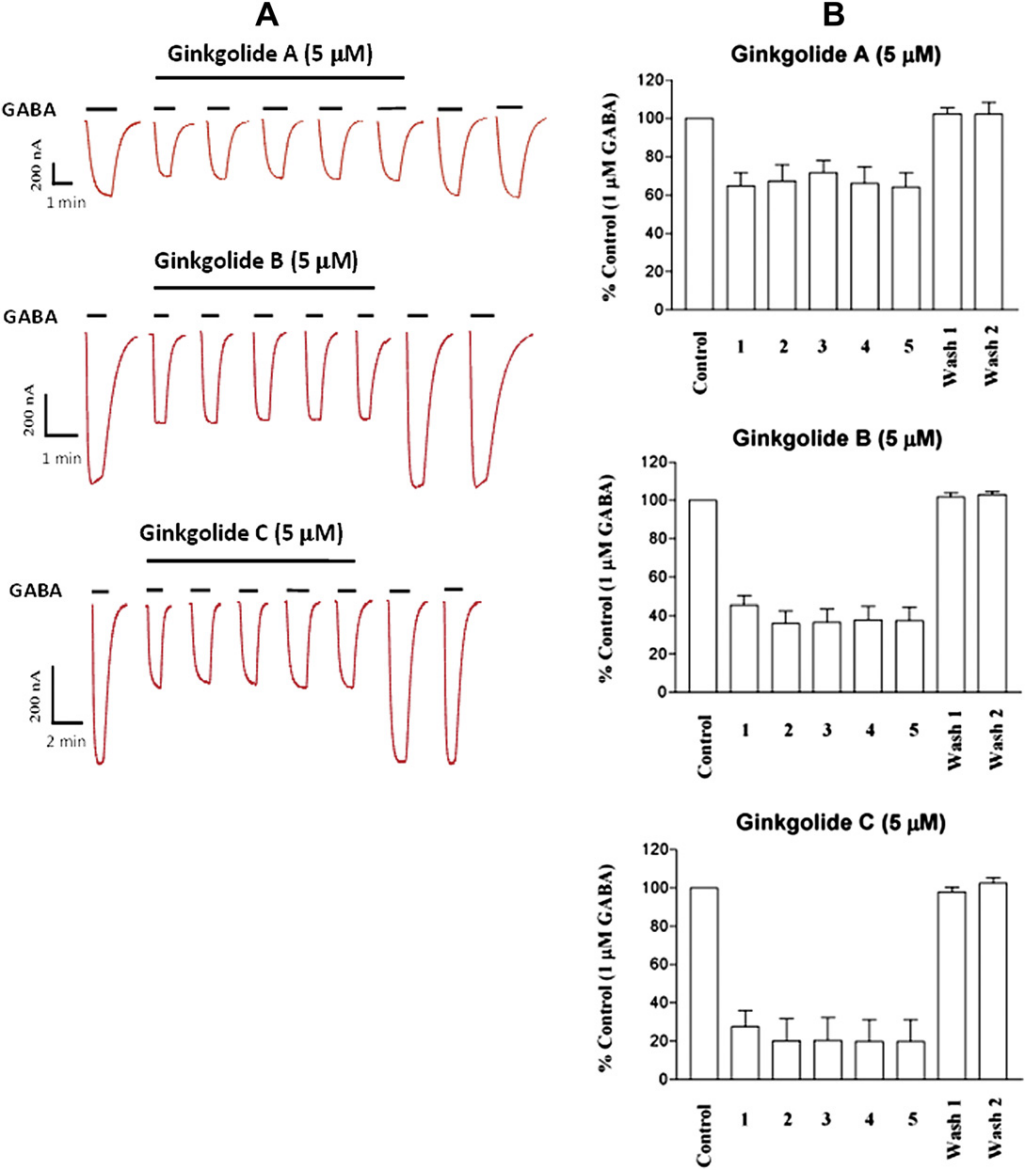
Use-dependent effect in the antagonism of ρ_1_ GABA_C_ receptors by ginkgolides. **A**. Examples of currents recorded during repeated co-applications of GABA and ginkgolides. The ginkgolides were applied 40 s prior to the first co-application with GABA and were continuously present until the fifth co-application. Each application was separated by a 3-min interval. **B**. Histogram representing the mean data of consecutive co-applications of GABA and ginkgolide A (*n* = 4), ginkgolide B (*n* = 6) and ginkgolide C (*n* = 4). The small increase in inhibition with the second application of ginkgolide B and C was not significant. Overall, there was no obvious evidence for use-dependent inhibition by the ginkgolides.

The use-dependence effects of ginkgolides A, B and C were examined by repeated co-applications with GABA (1 μM) during extended incubations of the ginkgolides (5 μM) following an initial control GABA response (Fig. 5). After 40 s preincubation with the ginkgolide, the set of five co-applications with GABA each showed a similar level of inhibition: ginkgolide A, 33.2 ± 1.4%; ginkgolide B, 61.3 ± 1.7%; and ginkgolide C, 78.4 ± 1.5%. This suggests that ginkgolide A, B and C are not obviously use-dependent.

**Fig. 6:**
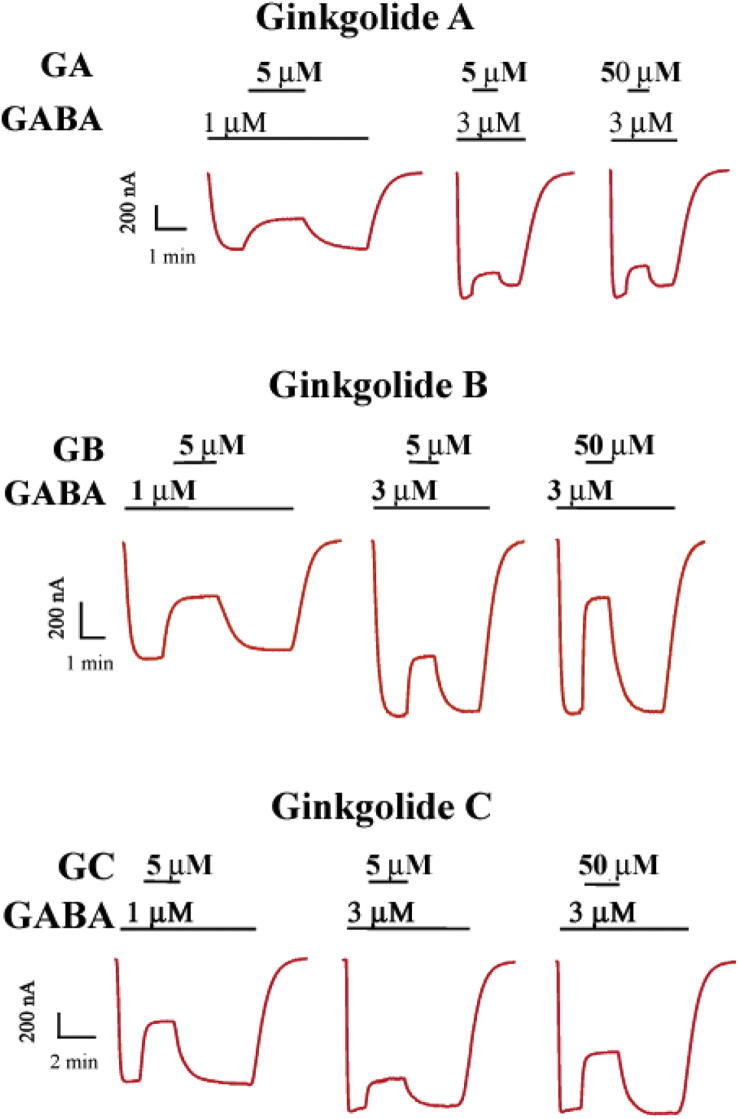
Recovery time from ginkgolide inhibition. Examples of current traces produced by the co-application of 5 or 50 μM ginkgolide A, B and C during the plateau phase of the response to 1 or 3 μM GABA. Note the faster recovery from block at higher GABA concentrations. The recovery times (10–90% rise times) are shown in Table 3.

Antagonism of the ginkgolides was reversible as indicated by full recovery of GABA responses following washout (Fig. 6). The recovery time from inhibition (measured as the 10–90% rise interval) was determined from the current responses obtained when ginkgolides A, B and C were co-applied during the plateau of the GABA-activated current (Fig. 6). The recovery times for inhibition by 5 and 50 μM ginkgolides A, B and C co-applied with 1 or 3 μM GABA are shown in Table 3. The results show that recovery of GABA responses from 5 μM gingkolide inhibition was faster at 3 μM GABA than at 1 μM, consistent with activation of the receptor in a concentration dependent manner. Increasing the ginkgolide concentration to 50 μM slowed the rate of recovery, suggesting the binding of ginkgolide favoured the closed states of the receptor.

### 3.4 Mechanism of action

**Fig. 7:**
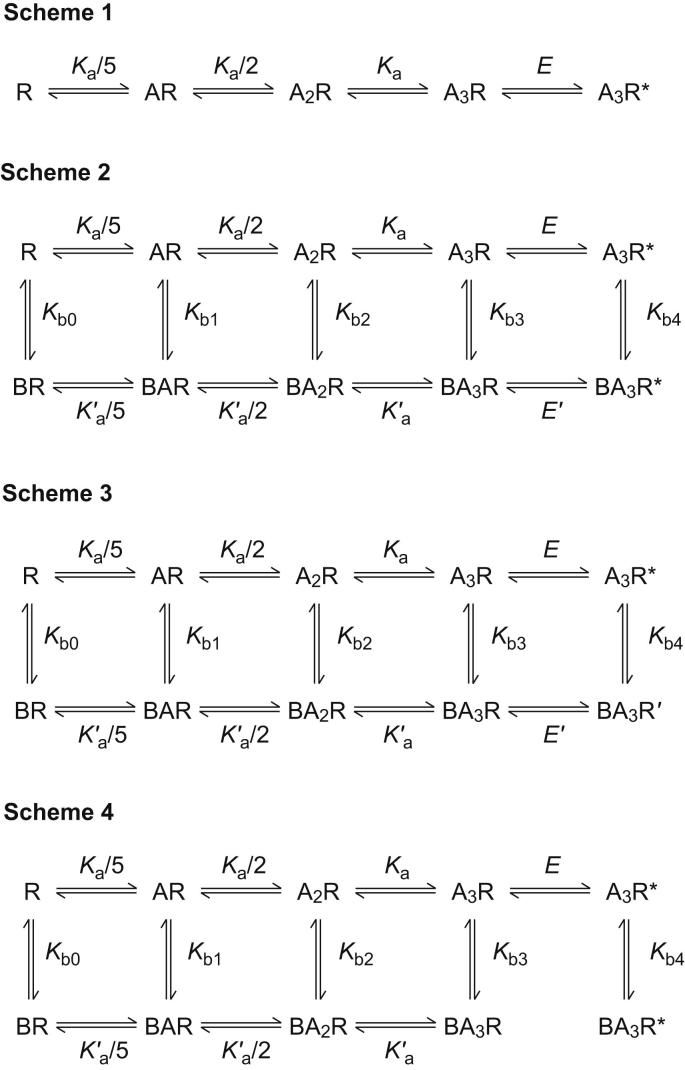
Macroscopic kinetic schemes fitted to equilibrium concentration–response curve data. Scheme 1 represents the sequential mechanism of Amin and Weiss (1996), which adequately describes the homomeric ρ1 GABA_C_ receptor function. This assumes five equivalent agonist binding sites but only three are required to be occupied to activate (open) the channel pore. All subsequent kinetic schemes are derivatives of the linear scheme, by incorporating different possible mechanisms for antagonism by ginkgolides. These are drawn from the cyclic mixed-antagonism mechanism described by Smart and Constanti (1986). Scheme 2 and scheme 3 both assume that ginkgolide binds to a single site in the receptor complex. The only difference between these schemes is that ginkgolide bound to the open state does not allow ion conduction in scheme 3 (BA_3_R′), while in scheme 2 the open state remains conducting (BA_3_R*). For scheme 4, the equilibrium between the fully-liganded closed and open states is removed, which allows for the possibility that the ginkgolide binds to a separate site in the closed state compared to the open state. In each case: R represents the receptor complex; A represents the agonist and the subscript the number of agonist molecules bound to the receptor; B represents the antagonist (blocker); the asterisk denotes the open (conducting) state of the receptor; *K*_a_ is the microscopic equilibrium constant for the agonist (GABA) and *K*_a_′ is the microscopic agonist equilibrium constant when ginkgolide is bound to the receptor (the macroscopic equilibrium constants are shown in each of the schemes); *K*_b0_ to *K*_b4_ are the equilibrium constants for the ginkgolide for each state of the receptor; *E* is the equilibrium constant for the opening reaction of the channel pore, where *E* = *β*/*α*, and *β* is the forward rate constant and *α* is the reverse rate constant; and similarly *E*′ is the equilibrium constant when ginkgolide is bound.

In an attempt to understand the mechanism of inhibition by ginkgolides at ρ_1_ GABA_C_ receptors, we began by fitting a sequential mechanism at equilibrium to the GABA concentration–response curves (in the absence of ginkgolides). The mechanism used (Fig. 7, Scheme 1) is that of Amin and Weiss (1996), where there are five equivalent agonist binding sites and three sites need to be occupied to activate the receptor, leading to a single open state. The fits to the GABA concentration–response data with this simple mechanism produced a good description of the data. However, the parameters obtained had large standard errors and there was strong dependency between the agonist equilibrium dissociation constant, *K*_a_, and the channel opening equilibrium, *E*, suggesting the data is not sufficient to precisely define each of the parameters. When the value for *E* was fixed to 11 (the value obtained by Amin and Weiss, 1996), the fits were visually indistinguishable to the free fits and the value for *K*_a_ was more reasonably determined as 3.22 ± 0.04 μM (value ± SE). This is of a similar order to the value of 1.4 μM obtained by Amin and Weiss (1996) and is consistent with the EC_50_ for GABA of 0.8 μM compared to 1.2 μM reported here. For all subsequent mechanisms fit to the data that incorporated inhibition by ginkgolides, the values for *K*_a_ and *E* were fixed to 3.2 μM and 11 (respectively), so as to provide better precision in estimating the other parameters.

**Fig. 8:**
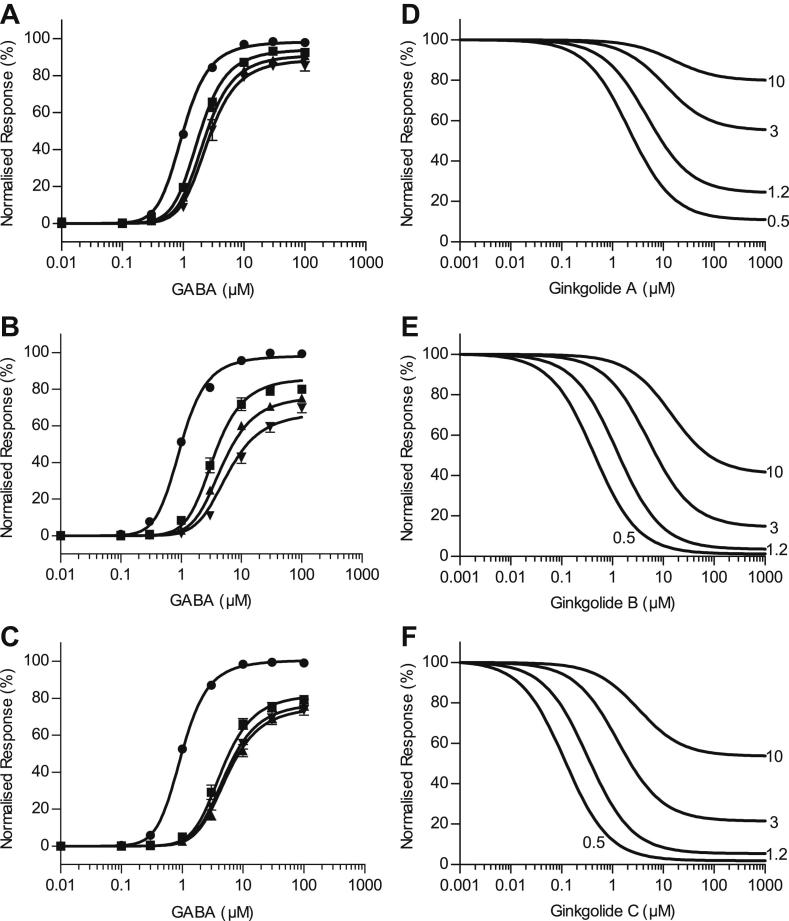
Results from scheme 2 (Fig. 7). Equilibrium concentration–response curves fitted with scheme 2 for (**A**) ginkgolide A, (**B**) ginkgolide B and (**C**) ginkgolide C. In each case, the parameters for the response to GABA alone were fixed (*K*_a_ = 3.2 μM and *E* = 11) to improve the precision on the remaining parameters estimated. All parameters for the fit were shared across the data sets for each ginkgolide with the exception of the concentration of the ginkgolide (0, 10, 30 and 100 μM). The fit of scheme 2 describes well the mixed-antagonism of the data sets for ginkgolide A, B and C. Panels **D**–**F** show the predicted inhibition curves generated with the parameters obtained from the fit to the concentration–response curves exhibit saturation of inhibition, where the bottom of the curve reaches a plateau rather than a full inhibition of the response. The plotted symbols in each panel are as for Fig. 3; equilibrium concentration–response data for GABA in the absence of ginkgolide (●) and in the presence of 10 μM (■), 30 μM (▴) and 100 μM (▾) ginkgolide.

Initially, a cyclic allosteric mechanism (Fig. 7, scheme 2) similar to that of Smart and Constanti (1986) was fit to the data. This scheme assumes a single binding site on the receptor complex that has an allosteric effect on all rate constants in the reaction mechanism. The scheme was fitted to the data allowing the agonist equilibrium constant, *K*_a_, to be related to the equilibrium constant in the presence of ginkgolide, *K*_a_′ by a constant ratio, *R* = *K*_a_/*K*_a_′. Similarly, the opening equilibrium constants *E* and *E*′ were related by the ratio, *S* = *E*/*E*′. This scheme fitted all three of the data sets well, describing both the rightward shift in the curves with increasing ginkgolide concentration and the decrease in the maximum response (Fig. 8A–C). The values obtained from the fits are shown in Table 4. As a test of the mechanism, the parameters obtained from the fit were used to predict the inhibition curves (Fig. 8D–F). The predicted inhibition curves exhibit rightward-shifts with increased GABA concentration and saturation of inhibition, where the bottom of the curves does not show complete inhibition for 3 and 10 μM GABA. Saturation of inhibition is a consequence of the allosteric mechanism, since the binding of antagonist alters the opening reaction equilibrium (*E*′), while the total number of receptors available to open remains the same. These predicted curves do not completely describe the experimental inhibition curves, as saturation was not generally observed except for ginkgolide B in the presence of 10 μM GABA.

**Fig. 9:**
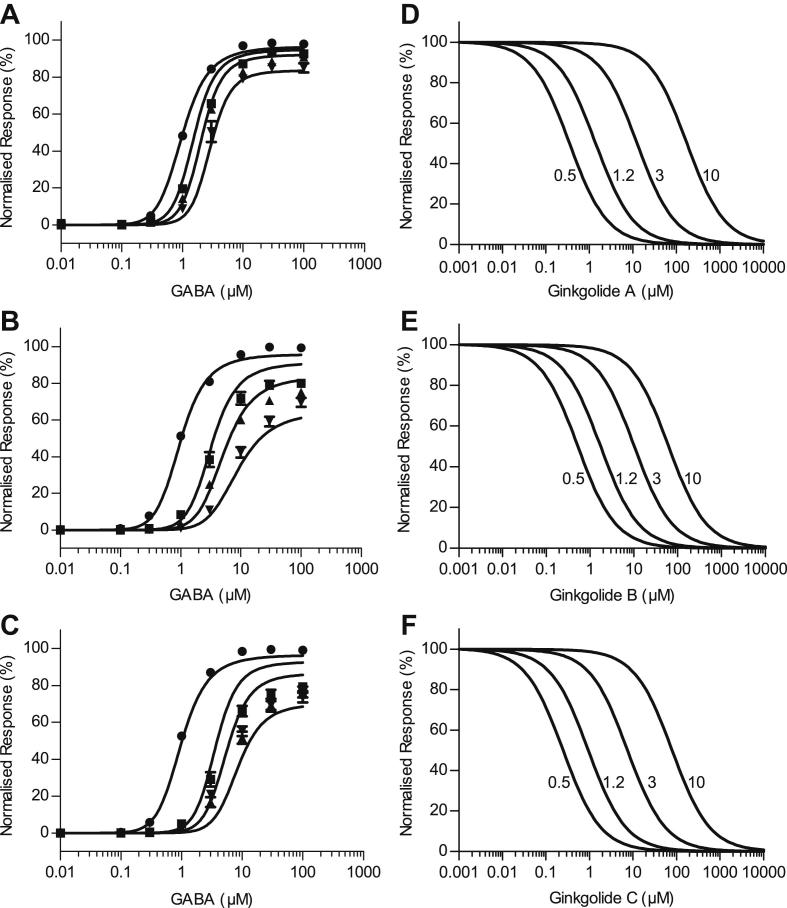
Results from scheme 3 (Fig. 7). Equilibrium concentration–response curves fitted with scheme 3 for (**A**) ginkgolide A, (**B**) ginkgolide B and (**C**) ginkgolide C. As for scheme 2, the parameters for the response to GABA alone were fixed (*K*_a_ = 3.2 μM and *E* = 11) and all other parameters were shared across the data sets for each ginkgolide with the exception of the concentration of the ginkgolide (0, 10, 30 and 100 μM). The fit with scheme 2 was noticeably worse, with the curve failing to follow the data points at the top of the concentration–response curve and consequently an increase in the standard deviation of the residuals for the fit. The predicted inhibition curves (panels **D**–**F**) exhibit complete inhibition at each of the GABA concentrations (numbers indicate 0.5, 1.2, 3.0 and 10 μM GABA). The plotted symbols in each panel are as for Fig. 3; equilibrium concentration–response data for GABA in the absence of ginkgolide (●) and in the presence of 10 μM (■), 30 μM (▴) and 100 μM (▾) ginkgolide.

If scheme 2 is modified so that ginkgolide bound to the open state of the receptor results in a non-conducting state (denoted as BA_3_R′ in scheme 3), then saturation of inhibition no longer occurs. As a result the predicted inhibition curves (Fig. 9D–F) are more similar to the experimental data, however the fit of this mechanism (scheme 3, Fig. 7) results in a much poorer fit to the top of the concentration–response curves (Fig. 9A–C) and there was strong dependency between the parameters *K*_b4_, *R* and *S*. As a result, some of the parameters (particularly for ginkgolide B) could not be adequately defined; they had large standard errors and there was an increase in standard deviation of the residuals for the fit compared to scheme 2 (Table 4).

In an attempt to find a mechanism that is able to fit the concentration–response curves and predict the inhibition curves, scheme 2 was modified to remove the channel opening equilibrium for the ginkgolide bound receptor (between BA_3_R and BA_3_R*; *E*′) to give scheme 4 (Fig. 7). This effectively means that ginkgolide is binding at two separate sites. This resulted in almost identical fits as scheme 2 for the concentration–response curves and still predicted inhibition curves with saturation. Scheme 4 is therefore functionally similar to scheme 2 with *E*′ having a value close to zero. Other more complicated schemes introduced further parameters, which meant there was increased dependency between the parameters and they could not be adequately defined.

### 3.5 Modelling and docking

**Fig. 10:**
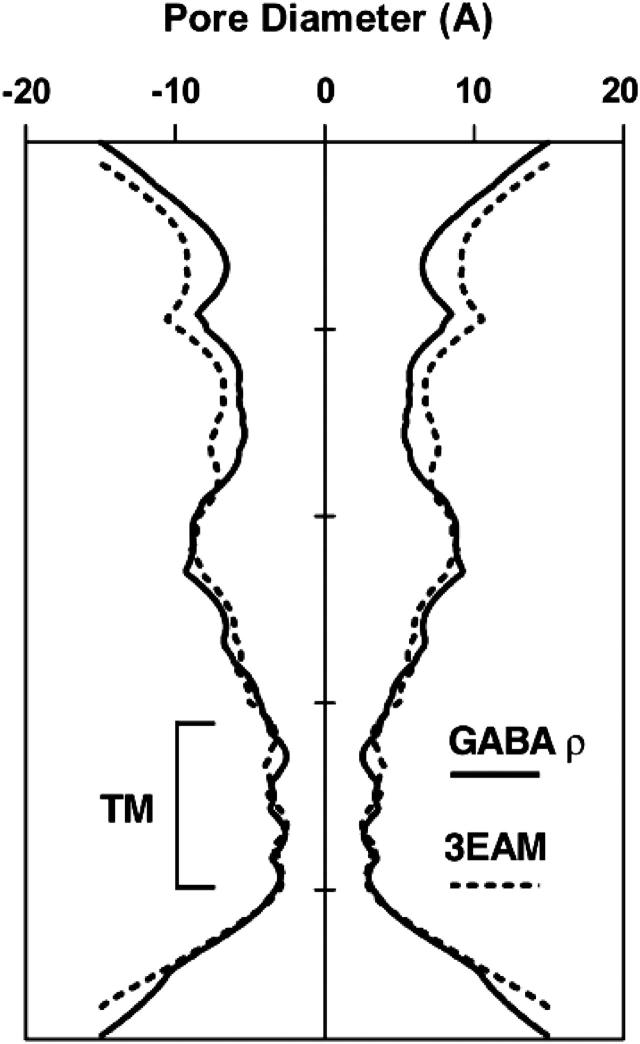
A comparison of the channel from 3EAM and the ρ_1_ GABA_C_ receptor homology model used in this study. The profile is plotted as the average pore diameter for the whole pentamer (solid line, ρ_1_ GABA_C_ receptor homology model; dotted line, 3EAM).

**Fig. 11:**
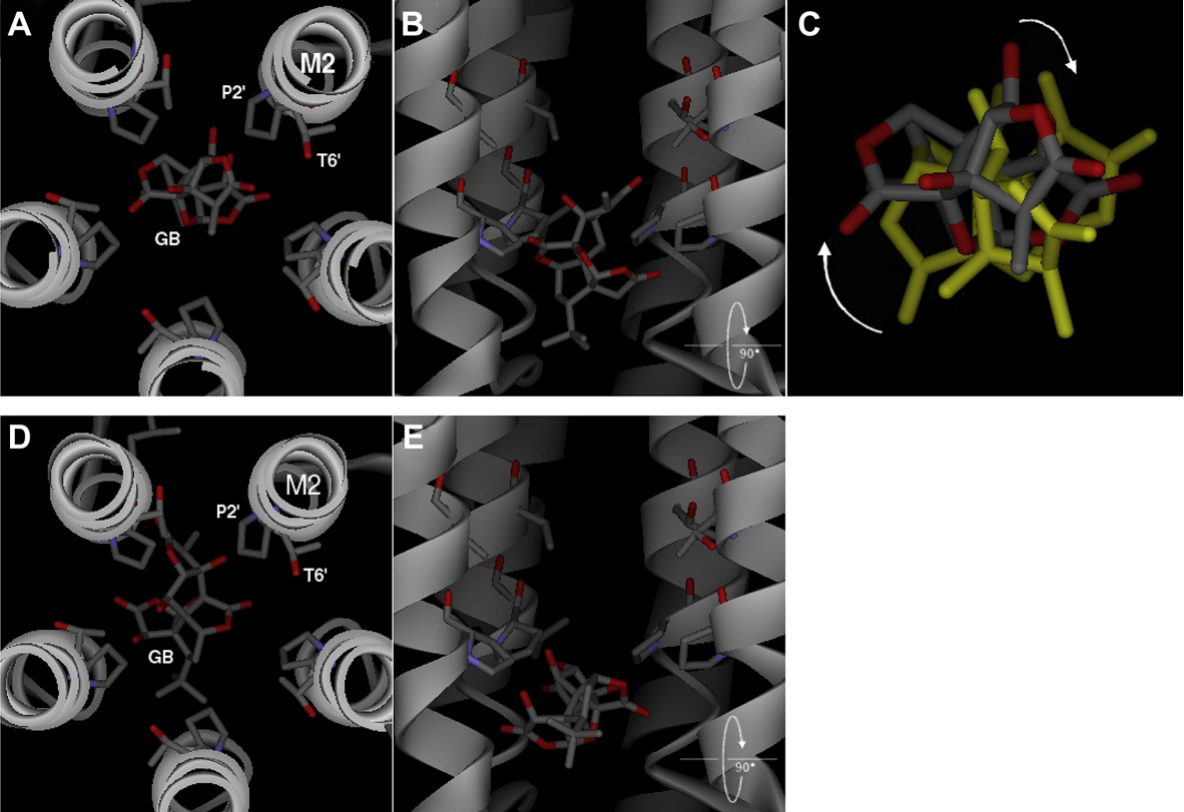
Examples of the two main docked poses for ginkgolide B in the ρ_1_ GABA_C_ receptor homology model. M2 helices are shown as ribbons, and apart from the side chains of residues P2′ and T6′, all others have been removed for clarity. For ginkgolide A, ginkgolide B (GB) and ginkgolide C, the orientations of the ligands were the same, and so only ginkgolide B (the most potent ligand) is shown. Panels A and D show the docked pose as seen looking from the extracellular domain, down through the receptor pore. Panels B and E are the same poses viewed from the side. Ginkgolide B is shown in stick representation. Panel C is in the same orientation as panel A, but shows an overlay of two poses, highlighting the rotation of the ginkgolide B ligand.

Amino acid sequence alignment of the ρ_1_ GABA subunit with the GLIC sequence showed a good conservation within the M2 pore region, and comparison of the average pore diameter for the ρ_1_ GABA_C_ receptor homology model and the 3EAM template of the GLIC receptor showed that the homology model was very similar within the transmembrane region (Fig. 10). Ginkgolides A, B and C docked in the pore between 2′ and 1′ residues. The docked poses fell into two major groups, either orientated along the axis of the pore (Fig. 11A and B) or, with the ligand perpendicular to the pore axis (Fig. 11D and E). A series of hydrogen bonds were predicted for all the ligands tested. In the majority (19/23) of the predicted H-bonds, this interaction was with the C3 or C10 hydroxyl of ginkgolides, A, B and C ligand. The unique C7 hydroxyl in ginkgolide C was predicted to interact in two of the poses, but the C1 hydroxyl in ginkgolide B and ginkgolide C did not account for any of the interactions.

## 4 Discussion

### 4.1 Ginkgolide antagonism

We show here that ginkgolide A, B and C are mixed antagonists at human recombinant ρ_1_ GABA_C_ receptors, exhibit potencies that are dependent on concentration of GABA, and require channel activation to dissociate from the receptor. The GABA concentration–response curves show approximate parallel shifts in the presence of ginkgolides, suggestive of a competitive effect. The noncompetitive effect is illustrated by the decrease in the maximum response of the GABA concentration–response curves in the presence of the ginkgolides. Apparent competitive antagonism is evident as IC_50_ values are clearly dependent on GABA concentrations. The ginkgolides are most potent at the lowest GABA concentration tested (0.5 μM) and the potency decreases as GABA concentrations increase (1.2 and 3 μM). A true noncompetitive antagonist would produce a constant IC_50_ value regardless of the agonist concentrations. The variation in potency with different GABA concentrations shows that the ginkgolides are neither classical competitive nor classical noncompetitive antagonists, confirming our results from GABA concentration–response curves in the presence of increasing antagonist concentrations. These results are indicative of the ginkgolides exhibiting mixed-type noncompetitive antagonism at ρ_1_ GABA_C_ receptors.

We have shown that bilobalide and PTX exhibit mixed-type noncompetitive antagonism at the ρ_1_ GABA_C_ receptors (Huang et al., 2006). Antagonism by PTX at GABA_C_ receptors has been shown to comprise both competitive and noncompetitive components (Woodward et al., 1992; Qian and Dowling, 1994; Wang et al., 1995; Qian et al., 2005; Carland et al., 2008). The competitive component could be removed from PTX antagonism at the ρ_1_ GABA_C_ receptor by substitution of the proline residue at position 2′ with the homologous residue of the GABA_A_ receptor α_1_ and β_2_ subunits and the glycine receptor α_1_ subunit at the saturated concentration of GABA but not at the EC_50_ of GABA (Wang et al., 1995; Carland et al., 2008). As PTX does not compete at the GABA binding site (Ramanjaneyulu and Ticku, 1984), the competitive antagonism of PTX is only apparent. This suggests that PTX acts as an allosteric inhibitor and alters the equilibrium for agonist binding and channel opening, consistent with different affinity of PTX for the receptor in the open and closed states from kinetic modelling of the GABA_C_ receptor (Qian et al., 2005).

Residues in −3′ to 2′ positions contributes to the GABA_C_ receptor pore (Filippova et al., 2004) and play a major role in channel gating in the Cys-loop receptors (Barry and Lynch, 2005; Miller and Smart, 2010). The residue at position 2′ has been shown to influence response kinetics, ion permeability, selectivity and conductance of the GABA_C_ receptors (Qian et al., 1999; Wotring et al., 2003, 2008; Carland et al., 2004a,b; Filippova et al., 2004; Zhu et al., 2007). Ginkgolide B has been shown by molecular modelling to be structurally similar to PTX (Ivic et al., 2003) and the ginkgolides only differ from one another in the hydroxyl substituents. In analogy to PTX, we suggest the ginkgolides also act as allosteric inhibitors altering the equilibrium for agonist binding and channel opening.

### 4.2 Use-dependence action of ginkgolides

We also examined the use-dependent actions of the ginkgolides on ρ_1_ GABA_C_ receptors using procedures described by Goutman and Calvo (2004) and Huang et al. (2006). Multiple co-applications of ginkgolides with GABA, in the continued presence of ginkgolide, exhibited no change in the level of inhibition (Fig. 5A and B). Thus, there is no obvious use-dependence in the antagonism of the ginkgolides. However, there is a clear dependency of the recovery time from the ginkgolide block on the agonist concentration (Fig. 6).

The association rate of GABA is four orders of magnitude slower than diffusion at the ρ_1_ GABA_C_ receptors indicating GABA has restricted access to its binding site at this receptor (Chang and Weiss, 1999). They also showed that GABA dissociates at two rates and proposed two mechanisms for dissociation of GABA. The faster rate corresponds to the true microscopic dissociation rate of agonist dissociation from the GABA binding site. The slower rate occurs because pore opening detains GABA release. That is GABA dissociation is inhibited during receptor gating. This helps to explain why the dissociation rate of PTX is faster than that of GABA at the GABA_C_ receptors in acutely isolated catfish cone horizontal cells (Dong and Werblin, 1996). In analogy to PTX, we suggest that the lack of use-dependent antagonism by ginkgolides at the ρ_1_ GABA_C_ receptor is because its dissociation rate is much faster than GABA. The slow association rate of GABA and a fast dissociation rate for ginkgolides could also account for the observed time course of recovery from ginkgolide block (Fig. 6) that is dependent on the GABA concentration.

### 4.3 Kinetic models of ginkgolide antagonism

Mixed-antagonism by PTX was first shown at lobster muscle GABA receptors (Constanti, 1978; Smart and Constanti, 1986). An allosteric model was developed where PTX interacts with the receptor complex in both channel closed and channel open states, and modulated the equilibrium constants between the receptor states (Smart and Constanti, 1986). This scheme accounted for the mixed-antagonism and the saturation of inhibition observed for PTX.

To explain the mechanism of action of ginkgolides at ρ_1_ GABA_C_ receptors, the concentration–response curves in the presence and absence of ginkgolides were fit with several kinetic schemes. All schemes assumed that there were five equivalent GABA binding sites on the receptor complex and that three binding sites needed to be occupied in order to activate the receptor with a single open state (Amin and Weiss, 1996). The data were best fit with schemes 2 and 4, based upon the cyclic kinetic scheme of Smart and Constanti (1986). Scheme 4 allows for the possibility that the ginkgolide binds to a different site in the closed and open states of the receptor. Both schemes 2 and 4 fitted the data equally well and predict a higher affinity for ginkgolides in the closed states. This is likely to account for the recovery from inhibition observed in Fig. 6 (rise times shown in Table 3).

In support of scheme 4, a kinetic model of the ρ_1_ GABA_C_ receptors indicated that the affinity of PTX was higher for the receptor in the closed than the open state (Qian et al., 2005). There is some evidence that PTX binds to a different site in the closed and open state of the receptor. PTX has been shown to protect the residue mutated to cysteine at positions 2′ and 17′ of the GABA_A_ α_1_ subunit from cross-linking with the sulfhydryl-reactive probes derived from the GABA_A_ noncompetitive blockers (Perret et al., 1999). PTX has been proposed to bind to a use-dependent site and a site independent of GABA at GABA_A_ and GABA_C_ receptors. Co-application of GABA with PTX generated an onset current transient from GABA_C_ receptor in acutely isolated catfish cone horizontal cells not pre-incubated with PTX but not in the cells pre-incubated with PTX (Dong and Werblin, 1996). The butyrolactone antagonist α-isopropyl-α-methyl-γ-butyrolactone (α-IMGBL) abolished the use-dependence part of the PTX block but had no significant effect on PTX block of GABA peak current at the GABA_A_ receptors in dissociated rat hippocampal neurons (Yoon et al., 1993). PTX has been shown to bind to the closed and the GABA-bound open states of the GABA_A_ receptors (Newland and Cull-Candy, 1992; Dillon et al., 1995). In parallel to PTX, we suggest that the ginkgolides may also bind to different sites of the ρ_1_ GABA_C_ receptor in the closed and open states, and that they bind with higher affinity to the receptor in the closed state.

### 4.4 Structure activity relationship of the ginkgolides

At ρ_1_ GABA_C_ receptors, the ginkgolides and PTX showed a significant decrease in potency with increase concentration of GABA (Table 2; Huang et al., 2006). Ginkgolide A is significantly less potent than ginkgolides B and C (Table 2) and much less potent than PTX (Huang et al., 2006). The variation between the potency of the ginkgolide A and ginkgolides B and C may be explained, at least in part, in terms of their structural differences. In the X-ray structure of ginkgolides B and C, there is an intramolecular hydrogen bond between the C1 and C10 hydroxyls (Fig. 1) (Dupont et al., 1986; Sbit et al., 1987; Zhao et al., 2002). The hydrogen atom of the C1 hydroxyl forming a hydrogen bond to the oxygen atom of the C10 hydroxyl is evident in the X-ray structure of ginkgolide C (Zhao et al., 2002). The ^1^H NMR resonances confirmed the presence of the intramolecular hydrogen bonding and also assigned the hydrogen bond donor role to the C1 hydroxyl and the acceptor role to the C10 hydroxyl in ginkgolides B and C (van Beek and Lankhorst, 1996; Bernet and Vasella, 2000).

The intramolecular hydrogen bonding between the C1 and C10 hydroxyl provides the rationales for the size of ginkgolide B (20 carbon atoms) being not so different when it was overlayed on PTX (15 carbon atoms) (Ivic et al., 2003). With the absence of the C1 hydroxyl, there is no intramolecular hydrogen bond to influence the structural conformation of ginkgolide A. All conformers of ginkgolide A have a larger distance-spanning lactones C and F than those of ginkgolide B (Ivic et al., 2003). This may account for the potency of ginkgolide A being distinct from that of ginkgolides B and C at ρ_1_ GABA_C_ receptors. The additional hydroxyl at C7 in ginkgolide C could hinder hydrophobic interaction of the *t*-butyl side chain to the pore residue, and may account for the lower potency of ginkgolide C compared to that of ginkgolide B.

### 4.5 Allosteric mechanism of action versus docking to pore residues

In ginkgolides B and C, the C1 hydroxyl acts a hydrogen bond donor in the intramolecular hydrogen bonding with the C10 hydroxyl (Dupont et al., 1986; Sbit et al., 1987; van Beek and Lankhorst, 1996; Bernet and Vasella, 2000; Zhao et al., 2002). This explains why the C1 hydroxyl was not predicted to hydrogen bond in any of the docking poses. Docking of the ginkgolides close to the proline residue at position 2′ suggests that the binding site of the ginkgolides may overlap with the PTX binding site. The residue at position 2′ has been suggested by site directed mutagenesis and molecular modelling to act as a secondary interacting residue to stabilize the binding of PTX to threonine at position 6′ at the homology model of GABA_A_, GABA_C_ and GABA_RDL_ receptors, and mutation of the residues at this position significantly affects the receptor sensitivity to PTX (Xu et al., 1995; Gurley et al., 1995; Perret et al., 1999; Zhorov and Bregestovski, 2000; Chen et al., 2006; Erkkila et al., 2008; Thompson et al., 2011, 2012). However, kinetic studies have shown that PTX acts by allosteric stabilization of GABA bound desensitized state(s) of the GABA_A_ receptors and not by directly occluding the pore (Twyman et al., 1989; Newland and Cull-Candy, 1992; Ramakrishnan and Hess, 2005; Krishek et al., 1996; Korshoej et al., 2010).

Mutations in the pore domain have been shown to affect picrotoxin affinity and gating by GABA, and also uncouple gating and desensitization of the GABA_A_ receptor (Buhr et al., 2001; Scheller and Forman, 2002). Thus, gating/desensitization being uncoupled to the pore domain could account for the change in PTX sensitivity of the receptors in response to mutation of the pore residues. Proline 2′ may constitute a part of the structure that couples gating and desensitization of GABA_C_ receptors as it has been shown to influence response kinetics, ion permeability, selectivity and conductance of the GABA_C_ receptors (Qian et al., 1999; Wotring et al., 2003, 2008; Carland et al., 2004a,b; Filippova et al., 2004; Zhu et al., 2007). As in the GLIC X-ray structure, the conformation in the homology model is likely to represent the open conformation. However, this may not be adequately represented in the conformation that binds the ginkgolides. This could explain the conflicting predictions of the molecular modelling and that of the fitted kinetic schemes that suggest the ginkgolides do not bind in the channel pore. Until we have a high-resolution structure of the GABA receptor, we must be cautious in the interpretation of the docking data, especially as the ρ_1_ GABA_C_ receptor has a proline residue at the 2′ position, which is not present in the template and has the potential to significantly influence the receptor structure. *Cis–trans* isomerization of proline has been shown to act as a switch in the opening of the pore of the 5-HT_3_ receptors (Lummis et al., 2005).

In conclusion, the ginkgolides exert mixed-type noncompetitive antagonism at ρ_1_ GABA_C_ receptors. We suggest that this is explained by an allosteric inhibition mechanism. Kinetic modelling predicts that ginkgolides exhibit saturation of antagonism at high concentrations of GABA, but this was only partially observed for ginkgolide B. It also suggests that there may be different binding sites in the closed and open states of the receptor, with a higher affinity for the receptor in the closed state.

## Figures and Tables

**Table 1:** Parameters estimated from the fit of the Hill equation to concentration–response curves in the presence and absence of ginkgolides.

Antagonists (Ant)		*n*	GABA EC_50_ (μM)	GABA + Ant EC_50_ (μM)	GABA *n*_H_	GABA + Ant *n*_H_	Max. GABA response (%)
Ginkgolide A	10 μM	5	1.02 ± 0.05	1.94 ± 0.09	2.03 ± 0.15	1.98 ± 0.12	94.78 ± 0.54*
	30 μM	5	0.90 ± 0.05	2.05 ± 0.09	2.29 ± 0.08	2.25 ± 0.04	91.44 ± 0.80†
	100 μM	5	1.05 ± 0.15	2.66 ± 0.37	2.09 ± 0.10	2.60 ± 0.22	85.66 ± 1.34*
Ginkgolide B	10 μM	6	1.04 ± 0.10	3.33 ± 0.42	2.03 ± 0.10	1.97 ± 0.10	80.65 ± 2.18*
	30 μM	5	0.94 ± 0.03	4.44 ± 0.20	1.97 ± 0.09	1.83 ± 0.06	75.12 ± 1.39*
	100 μM	5	1.07 ± 0.08	8.07 ± 0.61	1.68 ± 0.06	1.61 ± 0.05	69.56 ± 2.48†
Ginkgolide C	10 μM	4	1.06 ± 0.06	4.04 ± 0.33	1.86 ± 0.05	1.88 ± 0.05	79.24 ± 2.33*
	30 μM	4	1.09 ± 0.04	6.59 ± 0.36	1.81 ± 0.05	1.66 ± 0.05	76.50 ± 2.99†
	100 μM	6	0.99 ± 0.05	5.25 ± 0.31	1.38 ± 0.07	1.72 ± 0.03	73.44 ± 2.62*

EC_50_ is the concentration that evokes a response that is 50% of the maximum, *n*_H_ is the Hill coefficient and *n* is the number of data sets. Data are the mean ± S.E.M. Comparisons of the maximum responses in the presence and absence of ginkgolide were done by two-way ANOVA. †*P* < 0.05; *P < 0.01.

**Table 2:** Parameters estimated from fits of the Hill equation to inhibition curves.

GABA	Ginkgolide A			Ginkgolide B			Ginkgolide C		
	IC_50_ (μM)	*n*_H_	*n*	IC_50_ (μM)	*n*_H_	*n*	IC_50_ (μM)	*n*_H_	*n*
0.5 μM	5.74 ± 0.17	−0.99 ± 0.08	5	0.75 ± 0.07	−0.87 ± 0.05	6	0.83 ± 0.11	−0.75 ± 0.04	5
1.2 μM	42.3 ± 5.76	−0.82 ± 0.07	6	2.37 ± 0.22	−0.97 ± 0.07	7	5.39 ± 0.59	−1.04 ± 0.14	7
3.0 μM	181.6 ± 12.6	−0.61 ± 0.03	5	5.72 ± 0.63	−0.78 ± 0.06	5	22.0 ± 2.71	−0.81 ± 0.05	5
10 μM	873.7 ± 108.4	−0.73 ± 0.04	5	129.3 ± 21.6	−0.83 ± 0.06	7	519.8 ± 115.1	−0.83 ± 0.05	4

IC_50_ is the concentration that inhibits 50% of responses, *n*_H_ is the Hill coefficient and *n* is the number of oocytes from which data was collected. Data are the mean ± S.E.M.

**Table 3:** The percentage inhibition and recovery time from inhibition for co-application of ginkgolides.

	Inhibition (%)			Recovery time (s)		
[GABA]	1 μM	3 μM	3 μM	1 μM	3 μM	3 μM
[Ginkgolide]	5 μM	5 μM	50 μM	5 μM	5 μM	50 μM

Ginkgolide A	33.8 ± 3.4	15.9 ± 1.6	21.4 ± 1.9	48.4 ± 5.7	14.3 ± 4.6	18.4 ± 3.4
Ginkgolide B	55.5 ± 3.4	35.2 ± 6.8	54.6 ± 7.5	42.8 ± 8.6	21.3 ± 4.4	29.4 ± 3.8
Ginkgolide C	69.9 ± 11.3	33.3 ± 5.0	55.7 ± 7.6	52.9 ± 7.5	31.0 ± 5.3	42.0 ± 4.3

Data are the mean ± S.E.M. (*n* = 4–7 oocytes).

**Table 4:** Parameters estimated from the fit of schemes 2, 3 and 4.

		*S*_*y.x*_	Fitted parameters			Calculated parameters			
			*K*_b4_ (μM)	*R*	*S*	*K*_a_′ (μM)	*E*′	*K*_b3_ (μM)	*K*_b0_ (μM)
Scheme 2	GA	3.64	19.7 ± 3.9	0.56 ± 0.03	2.6 ± 0.2	5.7	4.3	7.7	1.3
	GB	4.55	35.2 ± 5.4	0.39 ± 0.02	8.6 ± 0.6	8.3	1.3	4.1	0.2
	GC	3.61	6.1 ± 1.1	0.38 ± 0.02	5.3 ± 0.2	8.4	2.1	1.1	0.1
Scheme 3	GA	4.46	611 ± 89	0.06 ± 0.05	0.09 ± 0.22	53.3	122	6789	1.5
	GB	5.65	1.7 × 10^11^ ± 2.3 × 10^17^	0.25 ± 0.01	1.0 × 10^10^ ± 1.4 × 10^16^	12.9	1 × 10^−9^	16.7	0.3
	GC	7.86	316 ± 132	0.10 ± 0.04	3.2 ± 4.1	31.2	3.4	98.7	0.1
Scheme 4	GA	3.64	19.7 ± 3.9	0.55 ± 0.03	–	5.7	–	7.7 ± 1.4	1.3
	GB	4.55	35.2 ± 5.4	0.39 ± 0.02	–	8.3	–	4.1 ± 0.5	0.2
	GC	3.61	6.1 ± 1.1	0.38 ± 0.02	–	8.4	–	1.1 ± 0.2	0.1

*K*_a_ was fixed to 3.2 μM and *E* was fixed to 11. *K*_a_′ and *K*_b0_ was calculated from *R* = *K*_a_/*K*_a_′ = *K*_b0_/*K*_b1_ = *K*_b1_/*K*_b2_ = *K*_b2_/*K*_b3_; *E*′ and *K*_b3_ were calculated from *S* = *E*/*E*′ = *K*_b4_/*K*_b3_. *S*_*y.x*_ is the standard deviation of the residuals from the non-linear regression. GA, ginkgolide A; GB, ginkgolide B; GC, ginkgolide C. Values are ±SE.
